# A Case of Lumbar Spinal Epidural Abscess and Facet Joint Septic Arthritis Caused by *Haemophilus influenzae* in an Immunocompetent Host

**DOI:** 10.3390/jcm14228006

**Published:** 2025-11-11

**Authors:** Yu-Mi Lee

**Affiliations:** Department of Infectious Diseases, Kyung Hee University Hospital, Kyung Hee University College of Medicine, Seoul 02447, Republic of Korea; cristal156@hanmail.net; Tel.: +82-2-958-8209; Fax: +82-2-968-1848

**Keywords:** epidural abscess, *Haemophilus influenzae*, facet joint septic arthritis

## Abstract

**Background:** *Haemophilus influenzae* rarely causes spinal epidural abscess or septic arthritis of the facet joints. We report a case of lumbar spinal epidural abscess and facet joint septic arthritis caused by *H. influenzae* in an immunocompetent host. **Methods**: A 53-year-old female patient with lumbar spine disc herniation presented with lower back pain 5 days before admission. **Results**: The patient was diagnosed with an epidural abscess at the right posterolateral aspect of the lumbar spine at the L4-5 level, as well as facet joint septic arthritis at the right L4-L5 and L5-S1 levels. The patient had no neurological deficit. On the 2nd day of hospitalization, the patient underwent decompressive laminotomy and posterior instrumentation. *H. influenzae* was identified in the blood cultures. She was prescribed intravenous ceftriaxone for 11 days until discharge and levofloxacin for 76 days after discharge. The patient recovered without neurological sequelae. **Conclusions**: This case represents the first report of septic arthritis of the facet joint and indicates that *H. influenzae* is a rare pathogen of spinal infection but can lead to a spinal epidural abscess, irrespective of the host’s immune status.

## 1. Introduction

*Haemophilus influenzae* is a Gram-negative bacteria, that can cause mild to serious infectious diseases. This species has two types; encapsulated (typeable) and unencapsulated (non-typeable). Among the six serotypes (a–f) of the encapsulated species, serotype b (Hib) comprised over 80% of invasive *H. influenzae* infections in the pre-vaccine era [1,2]. In the post-vaccine era, the incidence of Hib has dramatically decreased, whereas the incidence of non-typeable *H. influenzae* and serotype a *H. influenzae* has increased among individuals aged 65 years or older, often presenting with pneumonia [3,4,5]. Typeable *H. influenzae* infection often causes pneumonia, bacteremia, meningitis, septic arthritis, epiglottitis, otitis media, and purulent pericarditis, primarily in healthy children < 5 years of age. Non-typeable *H. influenzae* causes opportunistic infections, particularly in elderly individuals with predisposing medical conditions causing immuno-compression [2,6]. Invasive diseases less commonly develop by non-typeable *H. influenzae* [7]. Children younger than 5 years of age, adults older than 65 years, and people with specific medical conditions, such as aplenia, human immunodeficiency virus infection, immunoglobulin and complement deficiencies, malignancy, and sickle cell disease, are susceptible to *H. influenzae* infections [5]. Vaccine for *H. influenzae* type b is available; however, no vaccines are available for non-b or non-typeable *H. influenzae* infections [8].

Antibiotic resistance in *H. influenzae* has become a concern [9]. Resistance to β-lactam antibiotics such as ampicillin and amoxicillin is particularly increasing due to β-lactamase production [9,10]. Resistance related to changes in penicillin-binding proteins is also rising, especially in Japan, leading to increased resistance to ampicillin, amoxicillin, and many cephalosporins. Additionally, intrinsic efflux resistance is related to resistance of macrolides [9].

*H. influenzae* rarely causes vertebral osteomyelitis or epidural abscesses with only a few cases reported [11,12]. There were limited reports of spinal infections such as vertebral osteomyelitis and epidural abscess caused by *H. influenzae*. Qiao et al. reported a case of a cervical spinal epidural abscess caused by *H. influenzae* in an elderly patient with diabetes. There has also been a case report of spondylodiscitis with an epidural abscess in the lumbar spine caused by non-typeable *H. influenzae*. We present a case of spinal epidural abscess and facet joint septic arthritis caused by *H. influenzae* in an immunocompetent host. This study was approved by the Institutional Review Board (KHUH-2025-06-034) of Kyung Hee University Hospital, Seoul, Korea, which waived the need for written informed consent.

## 2. Case Presentation

**Figure 1:**
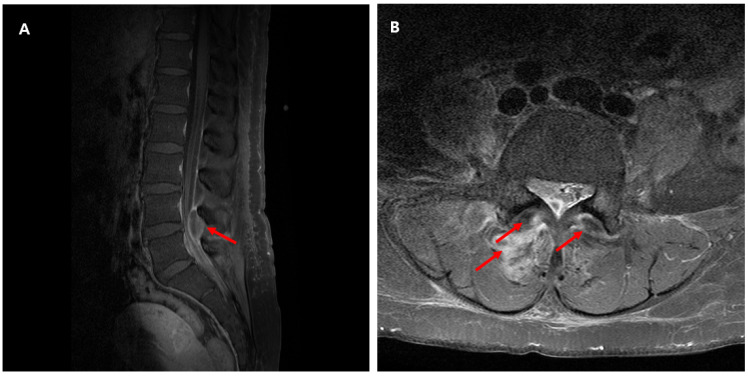
Lumbar spine magnetic resonance imaging (MRI). (**A**) Contrast-enhanced T1-weighted MRI Dixon water (**B**) T1-weighted MRI SPIR coronal. (**A**) Lumbar spine MRI showed epidural abscess (approximately 0.6 × 1.2 × 2.7 cm) at the right posterio-lateral aspect of the lumbar spine at the lumbar 4−5 level. (**B**) Infectious arthritis with capsular rupture of lumbar 4−5 level and lumbar 5-sacral 1 level of facet joints with periarticular abscess formation.

**Figure 2:**
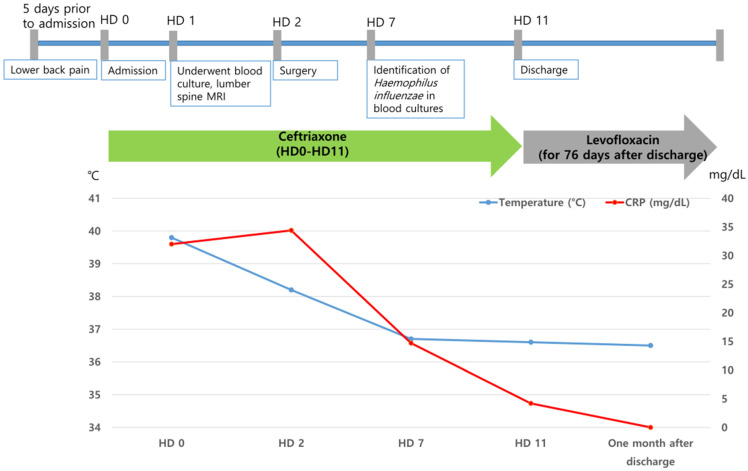
Clinical course and antibiotic treatment of a case patient. HD, hospital day; CRP, C-reactive protein; MRI, magnetic resonance imaging.

A 53-year-old female patient presenting with lower back pain 5 days before admission was admitted to the Infectious Disease Department of Kyung Hee University Hospital in the Republic of Korea in March 2025. She reported worsening pain in the right lower back, accompanied by throbbing pain extending to the back of the right thigh. The Numerical Rating Scale (NRS) for pain was 8 on the day of admission. The patient experienced chills before the onset of back pain. The patient had a medical history of lumbar spine disc herniation and mild intermittent pain. The patient did not take any medication. She had undergone an appendectomy 15 years prior. The initial vital signs were blood pressure, 115/60 mmHg; pulse rate, 116 beats/min; respiratory rate, 22 breaths/min; temperature, 38.2 °C, pulse oximetry, 95% on room air. Neurologic deficits were not observed in the neurologic examination. Laboratory examination revealed a white blood cell count of 14.86 × 10^9^/L (88.5% neutrophils) and a C-reactive protein was 32.0 mg/dL. Aspartate Aminotransferase (AST), Alanine Aminotransferase (ALT), and Alkaline Phosphatase (ALP) levels were slightly elevated at 37 IU/L, 63 IU/L, and 139 IU/L, respectively. The kidney function test results were within normal limits. Chest computed tomography revealed no abnormalities. Two sets of blood cultures were performed at the time of fever using BD Bactec Plus Aerobic/F and BD Bactec Plus Anaerobic/F bottles and a Bactec FX Instrument (Becton Dickinson, Sparks, MD, USA) on the day of admission. The patient underwent magnetic resonance imaging (MRI) of the lumbar spine. The MRI revealed an epidural abscess at the right posterior lateral aspect of the lumbar spine at the L4-5 level, measuring 0.6 × 1.2 × 2.7 cm, as well as infectious arthritis at the right L4-L5 and L5-S1 levels (Figure 1). The patient was initially prescribed with intravenous ceftriaxone (2 g per d). On the 2nd day of hospitalization, the patient underwent decompressive laminotomy and posterior instrumentation. Additionally, the patient underwent irrigation and drainage to remove the epidural abscess. On the day of surgery, two aerobic culture bottles showed culture-positive signals, and Gram-negative rods were observed. The culture-positive blood sample was sub-cultured on a Brucellar blood agar plate at 35 °C for 48 h under aerobic conditions. The obtained colonies were used for further identification using matrix-assisted laser desorption/ionization time-of-flight mass spectrometry (MALDI-TOF MS; Bruker Daltonik GmbH, Leipzig, Germany) with Biotyper software 2.3 and an MBT 6903 MSP library using a direct method. *Haemophilus influenzae* was identified. Antibiotic susceptibility testing was tested using the disc diffusion method. *H. influenzae* was susceptible to ampicillin, ampicillin/sulbactam, cefotaxime, ciprofloxacin, and trimethoprim/sulfamethoxazole. Since *H. influenzae* was susceptibility to ampicillin, β-lactamase testing was not performed. No bacteria were cultured from the four surgical specimens. The patient did not have a fever from the day after surgery. The lower back and right thigh pain gradually improved, except for occasional acute pain that occurred once or twice daily. On the 11th day of hospitalization, the patient was discharged without complications. The patient was administered levofloxacin (750 mg per d) for 76 days after discharge. Until one week after discharge, the patient reported improvement but still experienced throbbing pain in the right thigh and back pain. At the four-week follow-up, the patient reported no back pain or throbbing sensation in the leg, even without pain medication. Forty days after admission, the WBC count and CRP levels returned to within the reference interval range. The clinical course and antibiotic treatment of a case patient are shown in Figure 2.

## 3. Discussion

*H. influenzae* is a rare causative pathogen of vertebral osteomyelitis and spinal epidural abscess. We report a case of lumbar spine epidural abscess and facet joint septic arthritis caused by *H. influenzae*. *H. influenzae* infections spread through respiratory transmission via droplets, or through direct contact with droplets from symptomatic patients or asymptomatic nasopharyngeal carriers [13]. It is likely that nasopharyngeal colonization led to hematogenous spread, resulting in facet joint septic arthritis and an epidural abscess.

The case series from previous studies and this report on spinal infection caused by *H. influenza* are shown in Table 1. This case has several unique features. First, the patient, who was 53 years old, had no risk of *H. influenzae* infection. Previously only two reports of spinal epidural abscess caused by *H. influenza* were reported [11,12]. However, these cases occurred in elderly patients with diabetes. *H. influenzae* infections are more prevalent in immunocompromised hosts [14]. In particular, complement system deficiencies of C3 or C4 increase the risk of *H. influenzae* infections by weakening defense mechanisms, such as opsonization, bacterial clearance, and enhanced complement-dependent bactericidal activities [15]. However, *H. influenzae* infection can occur even in hosts with normal immunity according to our case.

Second, the spine epidural abscess is a rare infection site for *H. influenzae*. Cases of lumbar spine spondylodiscitis with epidural abscess and prosthetic hip joint infection caused by non-typeable *H. influenza* have been reported previously [12]. Recently, Luxi et al. reported a case of cervical spinal epidural abscess caused by *H. influenza* [11]. Cases of spine epidural abscess and vertebral osteomyelitis caused by *Haemophilus parainfluenzae* have been reported [16,17,18]. However, *H. influenzae* is a different species from *H. influenzae*. Although both belong to the *Haemophilus* genus, they differ in terms of pathogenicity, the presence of a capsule, and clinical presentation. *H. influenzae* is more pathogenic, particulary in children, than *H. parainfluenzae*. *H. parainfluenzae* typically lacks a capsule, which leads to its lower virulence, and often causes local infections rather than systemic infections, or is present as part of the normal flora.

Third, this is the first report of facet joint septic arthritis in an old woman caused by *H. influenzae*. John et at. reported on 117 cases of septic arthritis of the spinal facet joint [19]. *Staphylococcus aureus* is the most common pathogen causing septic arthritis of the spinal facet joint. However, *H. influenzae* has not yet been identified as a causative pathogen. Septic arthritis, such as that of the knee and shoulder, caused by *H. influenzae* is uncommon, but has been reported [20,21,22]. However, our case is the first report of septic arthritis of the spinal facet joint caused by *H. influenzae*.

Fourth, the case patient had undergone early surgery for a spinal epidural abscess. In general, not all cases of epidural abscess warrant surgical intervention. However, surgical treatment is recommended for better outcomes if there are neurologic deficits, lack of improvement with medical treatment, or risk factors such as Methicillin-resistant *Staphylococcus aureus* infection or diabetes [23]. According to the meta-analysis reported by Stratton et al., the failure rate of medical treatment for epidural abscesses is approximately 26% [24]. Delayed treatment of an epidural abscess can lead to irreversible neurological complications due to spinal cord compression, ischemia, or inflammatory mediators. Previous studies have identified risk factors for neurological sequelae in vertebral osteomyelitis as neurological deficits, epidural abscess, high inflammation levels, advanced age, and recurrence within 12 months [25]. Among patients with neurological deficit, the rates of neurologic sequelae differed according to the timing of surgery: 28% with early surgery, 55% with delayed surgery, and 66% without surgery. Early surgical intervention and timely drainage of the abscess can reduce neurological sequelae in patients with neurological deficits. Additionally, in terms of antibiotic treatment, a longer total treatment duration is associated with a lower recurrence rate within 12 months, so a sufficient antibiotic therapy duration is also emphasized to reduce the risk of recurrence. In previous case of epidural abscess caused by *H. influenzae*, the patient underwent evacuation surgery and achieved a good outcome, similar to our case [11,12]. Surgery for epidural abscess caused by *H. influenzae* may help rapidly relieve severe pain and reduce the duration of antibiotic use. The clinical outcome of epidural abscesses caused by *H. influenzae* has been favorable. There have been no reported cases of mortality, and patients typically showed improvement following surgery [11,12].

Our study has the limitation of not being able to determine whether *H. influenzae* is typeable or non-typeable strain. Based on epidemiological characteristics of *H. influenzae*, non-typeable strains are consistently the most prevalent *H. influenzae* that cause invasive disease, while Hib infections account for less than 20% of invasive cases [4]. Many studies have reported an increase in the incidence of invasive non-typeable *H. influenzae* infections [7,26]. From this perspective, it is reasonable to assume that the patient was infected by a non-typeable strain of *H. influenzae*. Despite this limitation, this report is valuable because it presents the first case of septic arthritis of the facet joint and documents the rare occurrence of a spinal epidural abscess caused by *H. influenzae*. Spinal epidural abscess and facet joint septic arthritis caused by *H. influenzae* rarely develop. Even in unusual cases, this case demonstrates the need to consider *H. influenzae* in the differential diagnosis to ensure prompt and appropriate treatment.

## 4. Conclusions

We present a case of spinal epidural abscess and infective arthritis of the facet joint caused by *H. influenzae* in an immunocompetent host. This case emphasizes that *H. influenzae* should be considered as a possible pathogen in spinal epidural abscesses even in immunocompetent adults, and that early surgical intervention may lead to excellent outcomes.

## Figures and Tables

**Table 1:** Case series of spinal infections caused by *Haemophilus influenzae*.

Case	Age/ Sex	Underlying Conditions	Symptom	Implant	Involved Site	Combined Infection	Co-Pathogen	Neurologic Deficit	Surgery	Antibiotic Therapy	Outcome	Neurologic Sequelae	Reference
1	F/79	Diabetes, hypertension, atrial fibrillation, history of right hip fracture (10 years ago)	Right hip pain Fever	Hip prosthesis	L4-5 Hip prosthesis	None	None	No	Yes	Cefotaxime (12 weeks) → Ciprofloxacin (21 days)	Survived	No	[12]
2	F/74	Diabetes, hypertension, disc at cervical spine	Neck pain Headache	No	C1-C2	None	None	No	Yes	Ceftriaxone (6 weeks)	Survived	No	[11]
3	F/53	Lumbar spine disc herniation	Right lower back pain with radiating pain in the right thigh Fever	No	L4-5	None	None	No	Yes (HD 2)	Ceftriaxone (11 days) → levofloxacin (76 days)	Survived	No	This case

F, female; C, cervical spine; L, lumbar spine; HD, hospital day.

## Data Availability

The original contributions presented in this study are included in the article. Further inquiries can be directed to the corresponding author.
